# Arterio-arterial graft as vascular access in hemodialysis, a case report and review of literature

**DOI:** 10.1016/j.ijscr.2024.110165

**Published:** 2024-08-13

**Authors:** Pezhman Kharazm, Sepehr Khajavi, Alireza Aghili

**Affiliations:** Clinical Research Development Center, 5 Azar Hospital, Golestan University of Medical Sciences, Gorgan, Iran

**Keywords:** Hemodialysis, Vascular access, Arterio-arterial graft, Case report

## Abstract

**Introduction and importance:**

Hemodialysis is the most prevalent type of Renal Replacement Therapy in end stage renal disease patients. Arterio-venous fistulas/grafts and central venous catheters are the most prevalent vascular accesses. But in some patients these options are not feasible because of different reasons. In such cases arterio-arterial grafts may be a viable option to provide vascular access. In this study we present a case of axillary artery arterio-arterial graft.

**Case presentation:**

A 66-year-old patient was scheduled for arterio-arterial graft implantation following failure of multiple previous vascular accesses. An 8 mm ringed PTFE graft was implanted in loop fashion subcutaneously in left anterior chest wall and anastomosed to transected ends of the axillary artery. Post-operative period was unremarkable and the graft was used for hemodialysis successfully for a period of 8 months.

**Clinical discussion:**

Arterio-arterial grafts can provide a flow rate up to 400 ml per minute for hemodialysis. Although the risk of steal syndrome is minimal in this form of vascular access, but the risk of limb ischemia following graft thrombosis should be considered.

**Conclusion:**

Arterio-arterial grafts may be the only available option for continued hemodialysis in some patients and any vascular surgeon should be familiar with this type of vascular access.

## Highlights

• The number of ESRD patients requiring hemodialysis is increasing everyday
• Arterio-arterial grafts are one of the last options to provide vascular success for hemodialysis
• Limb ischemia following graft thrombosis is one of the most important complications of arterio-arterial grafts
• Flow rates of more than 400 ml per minute could be problematic when the vascular access is an arterio-arterial graft

## 1 Introduction

The number of End Stage Renal Disease (ESRD) patients is steadily increasing every day [1]. Hemodialysis is the most prevalent type of Renal Replacement Therapy (RRT) among these patients [2]. According to the KDOQI guideline, the priorities for vascular access in patients requiring chronic hemodialysis are as follows: Arterio-Venous Fistula (AVF), Arterio-Venous Graft (AVG), and finally Central Venous Catheters (CVC) [3].

In some patients, due to various reasons such as heart failure, lack of suitable arteries or veins for fistula/graft, or their loss in previous surgeries, the possibility of implanting fistulas or grafts is not feasible, and these patients become dependent on catheters for hemodialysis. Among these patients, some who have managed to survive gradually develop stenosis/occlusion of the central veins, which eliminates the possibility of using central venous catheters as well [4,5]. Ensuring suitable vascular access in this group of patients is very challenging, and if possible, some of these patients are directed towards peritoneal dialysis. In such circumstances, Arterio-Arterial Graft (AAG) is one of the ways to provide vascular access for hemodialysis [6]. Few reports are present regarding this type of vascular access.

In this study, we present a case of ESRD on chronic hemodialysis underwent an AAG implantation for vascular access and review of existing information in this regard.

The study has been reported in line with the SCARE criteria [7].

## 2 Case presentation

The patient was a 66-year-old man on chronic hemodialysis for 7 years. He had a history of congestive heart failure and the implantation of AVF/AVG was impossible for him. So, during this 7 years, hemodialysis was done via catheters through the femoral, jugular and even subclavian veins on both sides. Gradually, with the occurrence of stenosis in the central veins, all of these accesses were failed. At the time of referral, the patient was dialyzing through a left femoral catheter, which had to be replaced due to thrombosis in the iliac vein.

Considering severe limitations in vascular access, the patient became a candidate for arterio-arterial graft implantation by a vascular surgeon.

On examination, the radial and ulnar pulses were palpable bilaterally, and there were no signs of arterial insufficiency. Arm pressure was 130/80 in right side and 125/80 in left side.

After careful pre-operative evaluation, the patient was scheduled for operation.

**Fig. 1:**
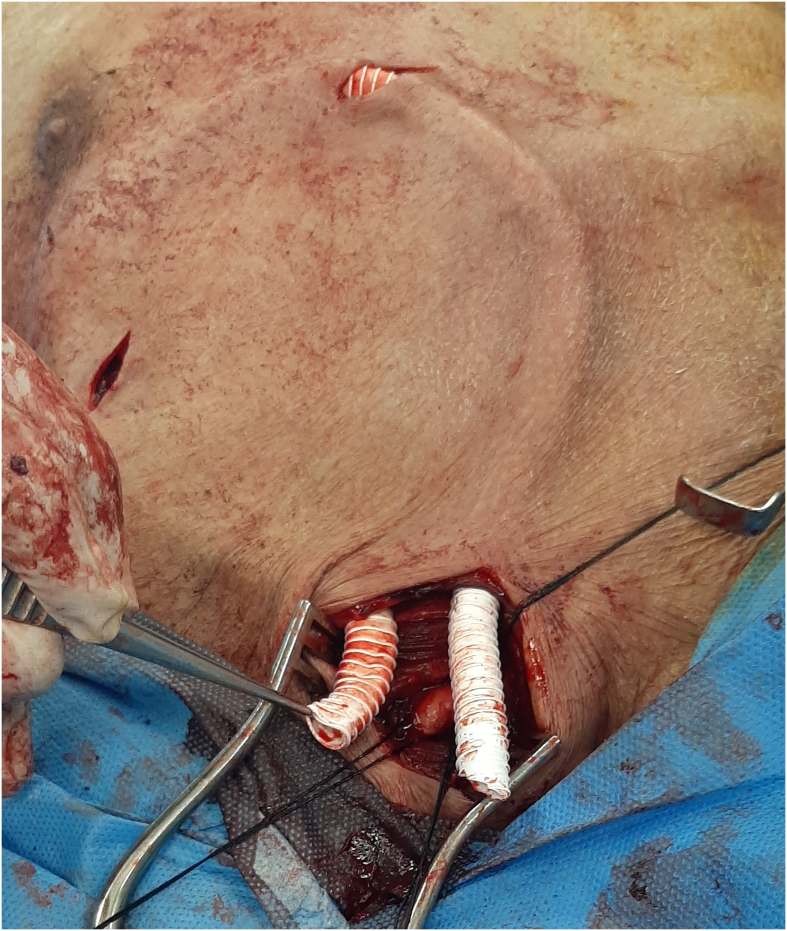
After exploration of the axillary artery, a piece of 8 mm ringed PTFE graft with sufficient length was passed through the subcutaneous tunnel as a loop.

Under general anesthesia, through an infraclavicular incision, the axillary arteries were explored and controlled (Fig. 1). To ensure independent limb perfusion from the axillary artery, after heparin injection, the axillary artery was temporarily occluded with a vascular clamp. Despite occluding the artery, the upper limb radial pulse remained palpable, although weakened. Pulseoximetery showed sinus waves with 99 % oxygen saturation.

**Fig. 2:**
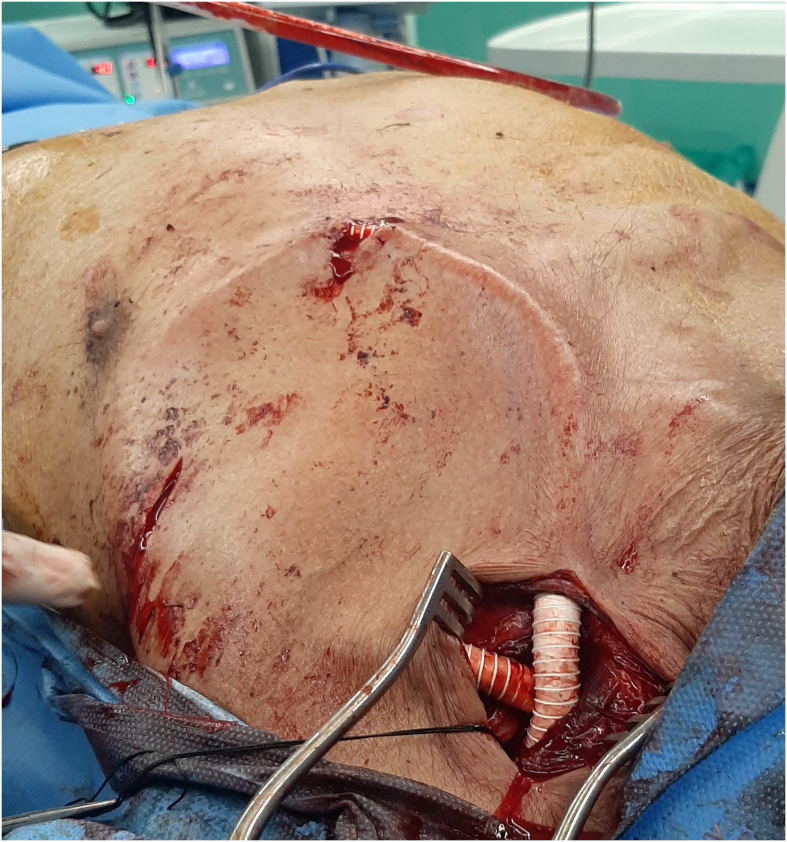
The graft was anastomosed to transected ends of the artery.

An 8 mm ringed PTFE graft was tunneled subcutaneously. The axillary artery was transected between two vascular clamps, and both ends were anastomosed to the graft with 6.0 polypropylene sutures (Fig. 2). Clamps were removed, hemostasis and arterial pulse distal to the graft were checked, and the incision was repaired. The patient was discharged in good general condition the day after surgery.

**Fig. 3:**
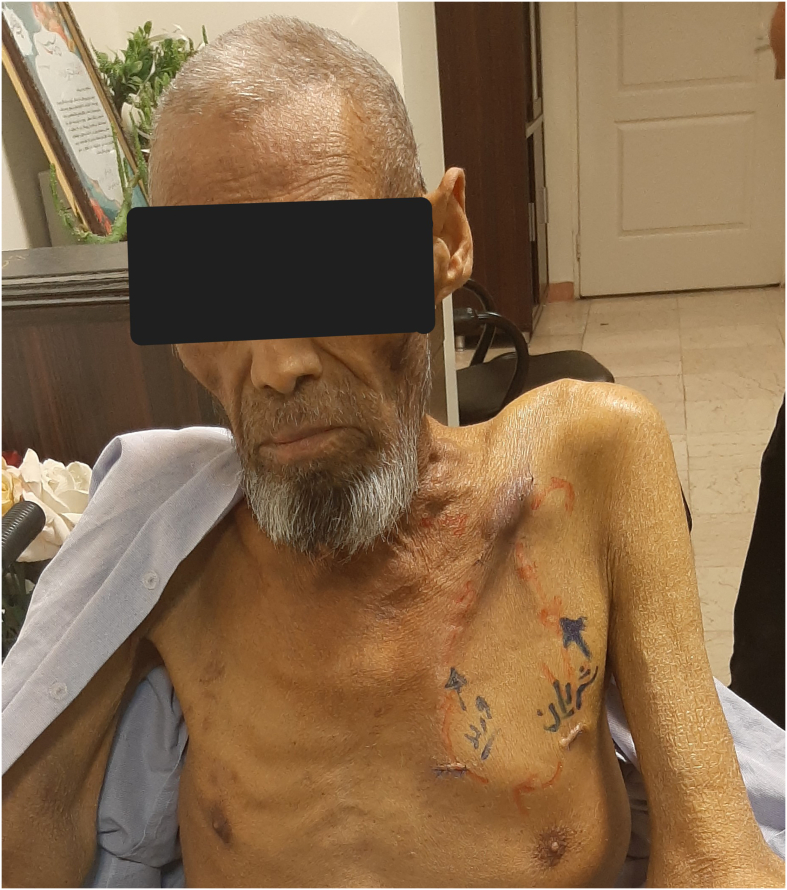
The graft was marked and permitted for access 2 weeks after operation.

Two weeks later, after suture removal, permission to vascular access from the graft was granted, and the femoral catheter was removed (Fig. 3).

The patient passed away 11 months later due to heart problems. During this period, there were no vascular access problem for hemodialysis, and the arteriovenous graft provided adequate flow for hemodialysis.

## 3 Discussion

Hemodialysis is the most common renal replacement therapy method in ESRD patients [2]. The arterio-arterial graft is one of the latest priorities for vascular access in patients needing hemodialysis. Bunger and colleagues first introduced this type of vascular access in 20 poor access patients. The indications for arterio-arterial graft placement in their study included heart failure, failed previous upper limb vascular accesses, and steal syndrome. They reported the risk of limb ischemia if the graft was thrombosed. Although, only one of four patients encountered limb ischemia following graft thrombosis which resolved following graft thrombectomy [8]. In our case, heart failure in addition to central venous stenosis following multiple catheter insertion was the indication for AAG.

In addition to the axillary artery, Zanow and colleagues also utilized the femoral artery in some of their patients for AAG, but ultimately they recommended the use of the axillary artery where possible [6]. We also preferred the use of the axillary artery for this purpose.

Although the use of arterio-arterial graft for hemodialysis in poor access patients has been defined for about 20 years, there are limited documented cases in literature, with only 151 cases recorded in 8 studies between 1997 and 2017 in a systematic review study conducted in 2018 [9]. In this systematic review, six-month primary patency rate of AAGs ranged from 67 % to 94.5 %. Additionally, it was indicated that the risk of limb ischemia was lower in patients who underwent end-to-side anastomoses compared to end-to-end interpositions [9]. In our study, end-to-end anastomoses were performed, and no signs of limb ischemia were observed during graft use. Doppler ultrasound performed once 6 months' post-operation confirmed graft patency and sufficient arterial flow.

Specific considerations for AAG compared AVG, besides the risk of ischemia following graft thrombosis, include: inability to administer medication through these grafts, the need for longer compression times at hemodialysis needle sites, and ultimately the limitation of flow rates to less than 400 cc/min. Higher flow rates can lead to limb pain, ischemia, and increased recirculation [10,11].

## 4 Conclusion

While arterio-arterial grafts are among the last options for vascular access in patients undergoing chronic hemodialysis, in some patients, they may be the only available option for continued hemodialysis. So, any vascular surgeon, as the main person responsible for providing different vascular access methods, should be familiar with technique and special considerations of this type of vascular access.

## Ethical approval

This study was approved by the Golestan University of Medical Sciences Research Ethics Committee with the following ethics code: https://ethics.research.ac.ir/IR.GOUMS.REC.1403.137.

Date of approval: 09/JUL/2024.

## Funding

There is no funding source for this study.

## Author contribution

Dr. Pezhman Kharazm, vascular surgeon and the patient's corresponding physician.

Sepehr Khajavi, Resident of General Surgery, literature review, article writing.

Alireza aghili, Resident of General Surgery, literature review, article writing.

## Guarantor

Dr. Pezhman Kharazm.

## Research registration number

0-0-58-114395, https://pajooheshyar.goums.ac.ir/main.

## Conflict of interest statement

No conflict of interest is present between authors.
